# The future of *Biomedical Digital Libraries*

**DOI:** 10.1186/1742-5581-3-5

**Published:** 2006-06-13

**Authors:** Marcus A Banks, Wayne J Peay

**Affiliations:** 1 Frederick L. Ehrman Medical Library, New York University School of Medicine, New York, NY, USA

Our colleague Charles J. Greenberg founded Biomedical Digital Libraries (BDL) in 2004, with the support of an outstanding team of editors and advisors [1]. He envisioned the journal as a "legitimate alternative to traditional specialty journals in the field, which have subscription fees and assumption of copyright by the publisher" [2]. Charlie has now decided to pursue other worthy endeavors, and remains on our editorial board. As the new editors-in-chief of BDL, we share his belief in the power of open access and in the potential of this journal.

BDL has covered a broad terrain. We have published resource reviews and commentaries, and documented innovative uses of technology that further scientific discovery and inform collection development. Readers are welcome to peruse all articles published thus far [3]. One strength of BDL is the variety of article types, including hypotheses, which are, "short articles presenting an untested original hypothesis backed solely by previously published results rather than new evidence" Hypotheses "should outline significant progress in thinking that would also be testable" [4]. We hope to publish the first hypothesis in BDL soon.

Print-only articles are not the only form of scholarly communication in the online era. BDL stands ready to publish multimedia works, including podcasts. Ramsey's "Multimedia Bootcamp" paper is the first BDL piece to feature movies [5], and we expect that many more will follow.

Regardless of the presentation format, our dedicated team of peer reviewers will critique submissions fairly and rigorously. After a healthy internal discussion BDL recently adopted an open peer review process, in which the authors and reviewers are all known to each other. Despite legitimate concerns that non-anonymity will cause reviewers to be less candid, we believe that transparency will lead to more thorough reviews overall. BioMed Central, BDL's publisher, has endorsed the concept of open peer review as well [6].

New presentation formats, new reviewing protocols - All of this causes us to think we are looking at a new type of journal. In an era of rapidly disseminated pre-print articles and frequently cited web log postings, the concept of the formal journal must evolve in order to remain vibrant. We believe that BDL is up to this challenge, and cordially invite the insights of our editors, advisors, authors and readers as we begin the journey.
